# Indian knowledge system plant based carbon dots: synthesis and optical sensing applications for environmental remediation

**DOI:** 10.1039/d6ra00662k

**Published:** 2026-05-26

**Authors:** Inderbir Kaur, Vandana Batra, Jasmina Baveja, J. Mejia, Y. Kumar, V. Agarwal

**Affiliations:** a Department of Electronic Science, Bhaskaracharya College of Applied Sciences, University of Delhi Delhi India; b Department of Physics, Bhaskaracharya College of Applied Sciences, University of Delhi Delhi India; c Invited Researcher at Center for Research in Engineering and Applied Sciences (CIICAp-IICBA), Autonomous State University of Morelos (UAEM) Av. Univ. 1001, Col. Chamilpa Cuernavaca Morelos 62209 Mexico; d Center for Research in Engineering and Applied Sciences (CIICAp-IICBA), Autonomous State University of Morelos (UAEM) Av. Univ. 1001, Col. Chamilpa Cuernavaca Morelos 62209 Mexico vagarwal@uaem.mx; e Departamento de Fisico Matematica, UANL Monterrey Mexico

## Abstract

One of the major problems in recent decades has been the absorption of harmful chemicals and toxins into the human body, either directly or indirectly. The excessive usage of heavy metals, like Cr(vi), Pb(ii), As(ii), Cd(ii), and organic contaminants such as pesticides, dyes, disinfectants, and food additives, and their untreated discharge by the industries, has had a profound effect on the environmental water systems as well as on the food chain. This ultimately impacts the human race. Carbon dots (CDs), a relatively new, cost-effective and sustainable nanomaterial, has recently gained attention as an alternative solution to address this issue, due to numerous benefits, such as additional value to waste, utilizing pollution-free resources and green processing procedures. Substantial type of precursors for green CDs have employed various plants from Indian knowledge system and have been used to detect metal ions and other organic contaminants in water. Some examples include tulsi leaves for detection of Cr(vi), mango peels for detection of the pesticide, mesotrione, *Aegle marmelos* (bael patra fruit) for detection of allura red, Fe(iii), and Cu(ii). The CDs synthesised using turmeric powder have been explored for degradation of acid azo dyes. It is well known that the ancient Indian knowledge system has consistently emphasized the role of nature in benefiting the human race. Vast Indian plant wealth is now finding alternate, sustainable ways to contribute towards the conservation of the environment. Thus, the Indian knowledge system may continue to act as a tool for protecting the environment and in turn the human race, using green nanotechnology.

## Abstract 2

Green carbon dots derived from Indian plant resources offer a sustainable strategy for detecting and degrading toxic heavy metals and organic pollutants in water systems.

## Graphical abstract

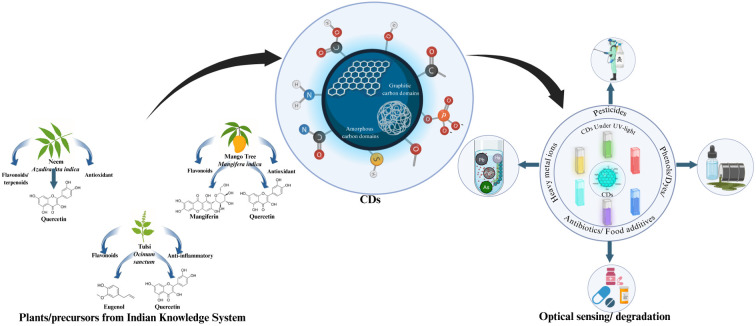

## 1. Introduction

One of the major issues humanity faces today is environmental contamination driven by the uncontrolled exploitation of resources of toxic pollutants into the environment.^1^ The textile sector primarily uses dyes, consuming over 80% of the total global dye production.^2^ The untreated release of these colouring agents into environmental waters is ecologically harmful. Colour obstructs the dispersion of sunlight, inhibiting photosynthesis, and restricting the development of aquatic life.^2^ Further, these dyes are highly poisonous, carcinogenic, non-biodegradable, and can also lead to numerous problems, including skin disorders and failure of the liver/kidneys.^2^ Additionally, the usage of heavy metals in mining, electroplating, and other industries is also a cause of major environmental pollution. Heavy metal contamination is detrimental to both the environment and public health due to its long-term harmful effects. As a specific example, excessive lead (Pb) exposure can harm the kidneys, and affect reproductive and nervous systems. On the other hand, mercury (Hg) toxicity results in kidney and neural disorders.^3^ Although iron is a required trace metal, its excessive levels can interfere with natural functioning and cause disorders such as hepatitis, arthritis, cancer, and heart failure.^4^ Additionally, antibiotics are widely employed as medicine in both human beings and animals to treat inflammation caused by bacteria.^5^ However, their excessive consumption and incorrect disposal can lead to the development of antibiotic resistance, digestive issues, and liver/kidney damage.^5^ Furthermore, the use of various food colorants and additives is a growing concern in developing nations, harming the environment and public health.^6^ Poor farmers have been reported to use malachite green (dye) to color vegetables, so that they appear fresh and green.^7^ These dyes are hard to remove from vegetables, even after being washed, and may end up inside the body and eventually the food chain, leading to various diseases such as heart/respiratory failure, and even cancer. They may also damage the spleen, liver, and kidneys.

Various conventional techniques being employed for remediation of dyes from effluents involve adsorption, coagulation/precipitation, redox mechanisms, ion exchange, ultrafiltration (reverse osmosis), flocculation and biodegradation,^8^ UV-light degradation, ozonation,^9^ advanced oxidation,^10^ electro/photo-chemical degradation^11^ methods, high pressure liquid chromatography, surface-enhanced Raman spectroscopy (SERS),^12^ enzyme-linked immunosorbent assay,^13,14^ magnetic molecularly imprinted polymers.^1^ Similarly, various analytical methods to identify heavy metals include X-ray fluorescence spectroscopy (XFS),^15^ atomic emission spectroscopy (AES),^16^ inductively coupled plasma-mass spectrometry (ICP-MS),^17^ atomic absorption spectroscopy (AAS),^18^ SERS and ion-selective electrodes.^19^ However, these techniques suffer from many drawbacks including costly/complex instruments with tedious pre-/post-treatments requiring proficient operators and the creation of toxic by-products, *etc.* As a result, these procedures are sparingly used.

In contrast to the conventional techniques mentioned above, fluorescence (FL) sensors, utilizing nanomaterials, are presently being used because of their increased selectivity and sensitivity.^20,21^ Although the primary focus has been on SnO_2_, CdTe, and CdS-based semiconductor quantum dot fluorescent investigations, the incorporation of toxic heavy metals in these sensors lead to significant environmental and health risks.^4^ Thus, designing metal-free, environmentally friendly sensors with high sensitivity is essential. In recent years, CDs have garnered substantial interest owing to their distinct characteristics, including facile surface functionalization, biocompatibility, and decreased toxic effects. They have been widely used for the detection of pollutants and other toxic chemicals due to their unique optical properties.^6^ Furthermore, plant-based precursors are being investigated as an alternate carbon source for forming CDs, rather than chemical precursors,^22^ due to their less toxicity, and being economically viable as well as environmentally sustainable.

## 2. Role of traditional Indian plants in environmental conservation

The ancient Indian knowledge system comprises of centuries old wisdom, which guides human beings to protect the environment, generate awareness about their surroundings including plants, animals and water bodies. The foremost human community to protect the environment was the Vedic community, where humanity was aware of its surroundings.^23^ In the Taittiriya Upanishad,^24^ a set of guidelines are provided to maintain a clean environment. The significance of existence of each creature for the survival of all other creatures has been illustrated by the Iso-Upanishad.^25^

Trees secrete essential oils and secondary metabolites in defense, from its various parts such as leaf, bark, fruit, stem, flower, roots, or needles, which are natural bioactive compounds rich in steroids, flavonoids, polyphenols, tannins, xanthan, alkaloids, and terpenes, *etc.*^26–30^ These compounds exhibit antioxidant, anti-inflammatory, antitussive, anti-diabetic, immunostimulant, antifertility, and anti-viral properties amongst others. Since ancient times, these bioactive compounds have been used in therapeutic applications^26,31^ for treating numerous ailments such as diabetes, cancer, kidney and liver disorders, asthma, cardiovascular-related problems, fungal and bacterial diseases^32,33^ and many more. The ancient Indian scientists documented a wide range of medications and treatments in Ayurveda and other ancient texts for maintaining good health and preventing diseases. Different plants are recognized on the basis of their characteristics and therapeutic advantages, which indicates our rich ancestral heritage and opulent knowledge of the plants' usage in daily life.

**Fig. 1:**
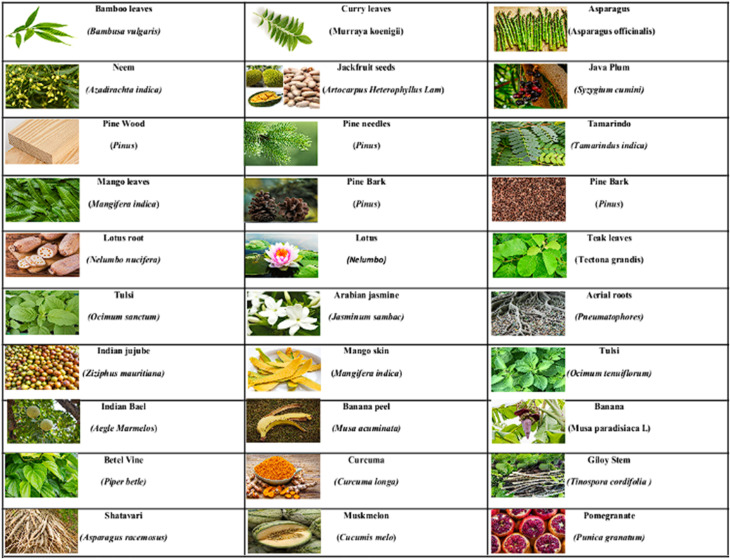
Some typical traditional Indian plants utilized in the synthesis of CDs.

Recently, the usage of Indian traditional plants has increased for therapeutics, food, and craft purposes due to their nontoxicity and less side effects. For example, Bael plant, is included in the ‘Climate Purifier’ plant category, due to its behaviour of absorbing harmful gases from the atmosphere as well as sinking toxic chemical pollutants.^34^ Mango, termed as the “National fruit of India” – is also known for its cardio protective, anticancer, anti-diabetic properties, *etc.*^33^ Lotus is adopted as “National flower of India” used for ornamental, nutritional and medicinal properties.^35^ Bamboo considered “Green Gold” is an inexpensive alternative to strong wood. It stores 1000 tonnes water, absorbs 12 tonnes of CO_2_ gas, and releases 35% higher oxygen gas as compared to the deciduous forest trees.^36^ Jackfruit is known as “giant nutrition fruit and a poor man's fruit”^37^ whereas *Asparagus racemosus* (Shatavari) is taken as ‘adaptogens’ for supporting the body to handle different types of stresses.^27^ The United Nations have declared *Azadirachta indica*, (neem) as “Tree of the 21st century” due to its versatile usage as a traditional Indian medicine for various diseases.^30^ Furthermore, these historic pieces of knowledge have inspired innovative research and improvements in health care and our surroundings. Fig. 1 shows some of the traditional Indian plants that have been used in the synthesis of CDs.

## 3. Synthesis and characterization of CDs using plant-based biomass

The fabrication of carbon dots from natural precursors represents a novel and eco-friendly approach in the diverse field of nanotechnology, employing accessible and environmentally sustainable materials, including vegetables, fruits and extracts from different parts of plants. Natural precursors provide a substantial carbon source and possess intrinsic biological compatibility, making them suitable for the synthesis of CDs with a reduced environmental impact.

**Fig. 2:**
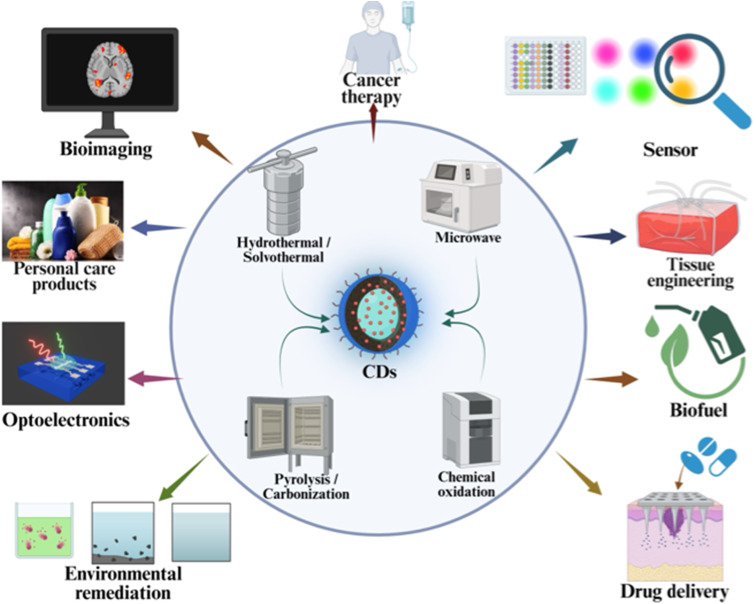
Some main green synthesis techniques (inner circle) and applications of CDs synthesized using natural precursors.

The synthesis of carbon dots (Fig. 2) predominantly employs a bottom-up approach, where the green precursors are carbonized either through hydro/solvo-thermal^38,39^ techniques or methods assisted by microwaves^40^ or ultrasonics. Hydrothermal/solvothermal synthesis involves the application of elevated temperatures and pressures to organic precursors in an aqueous/solvent solution, yielding CDs characterized by well-defined sizes and surface functionalization.^39^ Similar to this, microwave-assisted synthesis produces CDs quickly and effectively by irradiation with microwaves which escalates the carbonization process. Microwave synthesis is primarily attractive due to its capacity for rapid, energy-efficient heating, which results in reduced response times and enhanced control over morphology of particles.^41^ This synthesis procedure employs sources of carbon (glucose, citric acid,^42^ or other organic materials), forming their solution, and then subjecting microwave irradiation. Rapid dielectric heating results because of interaction of the microwaves with the polar molecules. This process ensures satisfactory product quality with little agglomeration by promoting homogeneous nucleation and development of CDs in addition to speeding up chemical processes. Apart from the concentration of the green precursor, the main variables that control the synthesis process are the duration and power of irradiation. The ease of application and scalability of microwave-assisted synthesis make it a noteworthy approach.

The use of ultrasonic waves represents an innovative method in nanotechnology, utilizing the distinctive advantages of ultrasound-assisted technique. Using high-frequency sound waves, the ultrasonic approach for CD synthesis creates vapor filled voids/bubbles in water/solvent containing green precursors. The collapse of these bubbles helps in creating high pressure & temperature locally, which promotes the formation of nanosized carbon particles from the fragmentation of carbon. This procedure offers advantages such as simplicity, cost-effectiveness, environmental sustainability, facilitating scalability, and high efficiency for producing CDs with adjustable characteristics.^43,44^ Several important parameters, including temperature, sonication power, and sonication time, can be optimized to greatly increase the yield of CDs produced using ultrasonic waves. On the other hand, in conventional pyrolysis, carbon source materials are thermally broken down to create CDs using temperatures, usually between 300 and 1000 °C, in an oxygen-free atmosphere.^45^ In this process, organic molecules experience carbonization and cracking, leading to the formation of carbon core and some functional groups on the surface (depending on the temperature). Fig. 2 depicts various synthesis methods of CDs using natural biomass and their main applications (colorimetric/optical sensing/photocatalytic degradation/electrochemical sensing *etc.*).

**Fig. 3:**
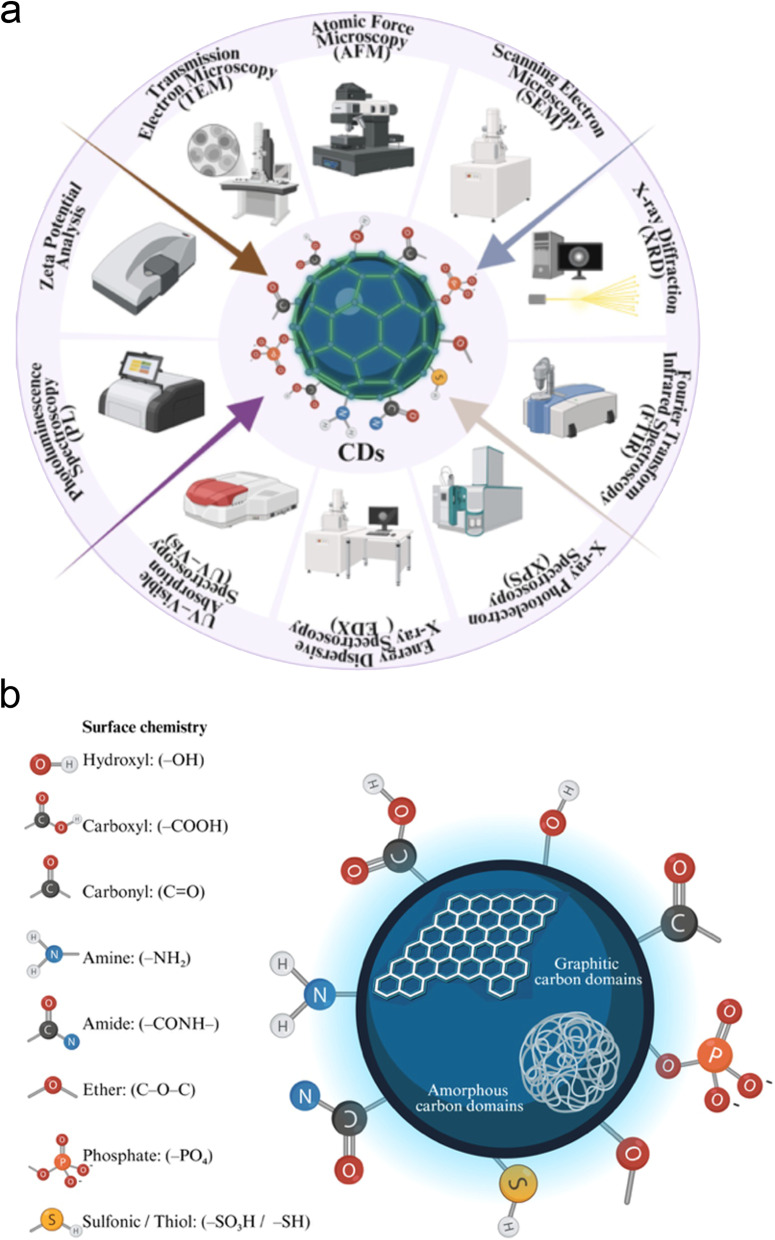
(a) Schematic representation of some common characterisation techniques employed for CDs. (b) Typical functional groups associated with doped and undoped CDs.

Fig. 3 shows the main characterization techniques used for the analysis of carbon dots (CDs), which enable the correlation of their physicochemical properties with their performance in various applications. Morphological and structural characteristics are generally evaluated by high-resolution scanning (HRSEM)/transmission electron microscopy (HRTEM). These techniques are used for finding the particle size, shape, and the degree of aggregation of CDs, with high resolution. In particular, TEM is widely used to observe the internal structure and possible crystalline domains, as well as to estimate the average size at the nanometric scale. On the other hand, SEM provides complementary information on surface morphology and the distribution of nanoparticles in different matrices.^46–48^ Additionally, Atomic Force Microscopy (AFM) enables the analysis of surface features and the measurement of the height of CDs, which is especially useful for confirming their quasi-spherical nature and their vertical dimension, providing three-dimensional information at the nanometric scale.^49^

The crystalline structure and degree of ordering of CDs are studied using X-ray diffraction (XRD), a technique that allows the identification of graphitic or amorphous structures, as well as the estimation of parameters such as interplanar spacing.^46,48^ Furthermore, the chemical composition and the nature of functional groups on the surface are determined by infrared spectroscopy^3^ (FTIR) and X-ray photoelectron spectroscopy (XPS). FTIR enables the identification of characteristic vibrational modes associated with specific bonds/groups such as O–H, C=O, C=S, C–O, –NH_2_, –COOH, evidencing the presence of oxygen and heteroatoms that influence the solubility and reactivity of CDs. Complementarily, XPS provides detailed elemental composition and the presence of oxidation states of the atoms, allowing for a more detailed analysis of surface chemistry and doping mechanisms.^46,50^

The optical characteristics of CDs are analyzed using UV-Vis and photoluminescence (PL) spectroscopy, which are fundamental techniques for understanding their light absorption and emission mechanisms. UV-Vis spectroscopy allows the identification of characteristic electronic transitions, such as π–π* and n–π* transitions, associated with conjugated structures and surface functional groups.^3,46,48,50^ Meanwhile, PL provides information on electron recombination processes and emissive centers, being particularly relevant for evaluating phenomena such as excitation wavelength-dependent emission, quantum yield, and the influence of surface defects.^50,51^ Likewise, zeta potential is used to evaluate the colloidal stability and surface charge of CDs in dispersion. This parameter is crucial for understanding electrostatic interactions between particles, as well as their behavior in aqueous media and biological systems, directly influencing their aggregation, dispersibility, and applications in sensing or bioimaging.^46,48,50^

Overall, the integration of these characterization techniques enables the establishment of a comprehensive correlation between the structure, surface chemistry, and optical properties of CDs.

## 4. Formation mechanism of CDs

The design of novel carbon dots with the required properties demands a deep understanding of their formation mechanism. Due to the simultaneous involvement of several variables, including reaction time, temperature, and precursor structure, it is an intricate task that requires systematic studies. In the detailed work from Rigodanza *et al.*,^52^ it has been shown that the formation of carbon dots consists of four different stages, involving aggregation, shell formation, collapse, and aromatization.

Similarly, the formation mechanism of CDs *via* the bottom-up technique has been shown to involve the carbonisation of small organic matter. This is achieved through a number of steps, including condensation, polymerisation, carbonisation followed by surface passivation.^53^ The tiny molecules go through the condensation processes like aldol condensation,^54^ Schiff base condensation,^55^ amidation^56^ and radical reaction,^57^ leading to the formation of chain-like intermediaries, which further lead to the creation of polymer like CDs.^58^ The polymers subsequently get carbonised, generating the carbon core, particularly at high temperatures.^59,60^ Furthermore, the remaining precursors, serving as surface passivating agents, can be altered on their surface to increase the luminescence of CDs. Research on doped and hybrid CDs, such as N, S-co-doped CQDs and Gd-doped CQDs, demonstrates that nuclei formation, growth, and carbonisation are significantly influenced by temperature and time^61,62^

**Fig. 4:**
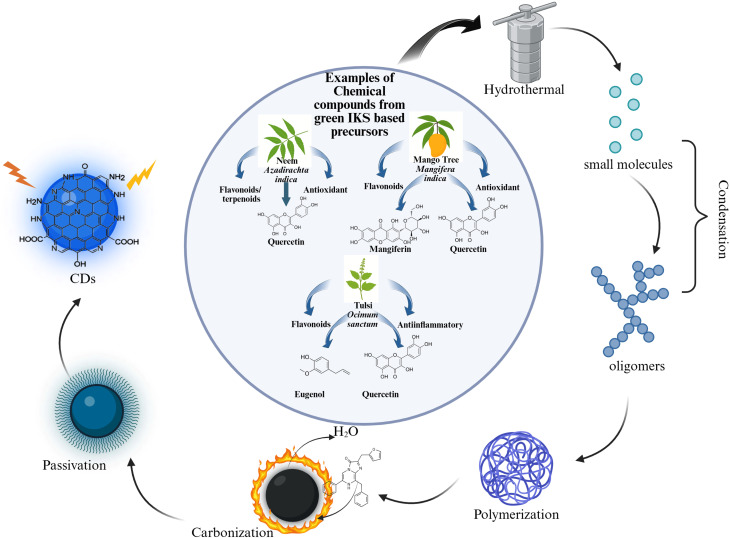
Schematic representation of the possible formation mechanism of CDs showing different stages.

Sustained microwave irradiation encourages intramolecular dehydration and C–C bond creation in palm kernel shell-derived CDS, generating aromatic functional groups and facilitating CD nucleation. The most acceptable formation mechanism is depicted in Fig. 4. Some specific examples of the precursors from IKS, along with their corresponding medicinal value as per the ancient texts and bioactive compunds are given in Table 1. The synthesis parameters and some resulting characteristics are also listed.

**Table 1:** Optimized synthesis conditions and results obtained from various characterisation studies in plant (from IKS)-based biomass. The functional groups and chemicals present have been specifically highlighted

S. no.	Precursor/plant family	Medicinal/general usage	Bioactive compounds present	Synthesis method/optimised synthesis conditions (temperature/duration/pH)	Dopant used	Characterisation techniques	Size (nm)/zeta potential	Functional groups reported
01	Curry leaves extract^3,26^(*Murraya koenigii*)/Rutaceae	Anti-bacterial, anti-inflammatory, anti-dysenteric, anti-diabetic, carminative and digestive properties	Vitamin C & A, essential oils α-terpinene sabinene, α-pinene, β-pinene, iron, phosphorus, magnesium oxalates, terpenoids, alkaloids, fiber	Microwave/800 W for 8 min	—	UV-Vis spectroscopy, FTIR	—	–OH, C=C, C=O, C–O, C–H, COOH
02	Giloy stem^28,46^ (*Tinospora cordifolia*)/Menispermaceae	Chronic fever, dengue fever (increases platelet count), virus infection, hay fever, improve digestion, boost immunity, reduces stress and anxiety, treats arthritis/gout, leprosy, anorexia, jaundice, diuretic, astringent, itching, bleeding piles, erysipelas, memory booster	Steroids, alkaloids, polysaccharides, glycosides, terpenoids, and lignans	Hydrothermal/160 °C for 5 h, pH = 5.6	Alkaloids (N source), terpenoids (C source), steroids (S source)	TEM, DLS, XRD, XPS, FTIR, zeta potential, UV-Vis & fluorescence spectroscopy, time-resolved fluorescence (lifetime)	3–7/−10.7 mV	C=C, C–H, C–C, C–N–C, C–O–C, C=O, N–C_3_, N–H, –NH_2_, C–S, S=O, C=S, –OH, COOH, COSH
03	“Shatavari” *Asparagus racemosus* root^27,48^ (Asparagaceae)/(Liliaceae)	Described as ‘rasayana herb’; anti-lung cancer activity, phytoestrogenic, anti-bacterial, cardio protective, anti-dyspepsia, anti-diarrhoeal, adaptogenic, antiaging, promoting intellect, physical strength, immunoadjuvant, used for neurodegenerative disorders	Steroidal saponins (shatavarin I–IV), quercetin, rutin, hyperoside, diosgenin, quercetin-3, glucuronide	Microwave/900 watts at 10 min	—	TEM, XRD, UV-Vis & fluorescence spectroscopy, FTIR, zeta potential	8/−48.4 mV	–OH, –COOH, C=O, C–H, N–H
04	Neem seeds (*Azadirachta indica*^50,63^)/Meliaceae	Biopesticides insecticidal, fumigant, manure, urea coating agent, soil conditioner, anti-viral, anti-fungal, anti-bacterial	Limonoids group (triterpenoids) including nimbin, azadirachtin, nimbidin, salannol, salanin, gedunin, quercetin	Hydrothermal carbonization/180 °C for about 12 h	Aqueous NH_3_ (as N source)	HR-TEM, SAED, XRD, XPS, FTIR, EDS, Raman spectroscopy, UV-Vis spectroscopy, fluorescence spectroscopy, zeta potential, TGA	2.5/−20.4 mV	–OH, –NH_2_, –C=C, C–N, NH, –COOH, C=O, C–O–C
05	Neem leaves^30,64^ (*Azadirachta indica*)/Meliaceae	Biopesticides, insecticidal, fumigant, manure, urea coating agent, soil, conditioner, anti-viral, anti-fungal, anti-bacterial	Limonoids group (triterpenoids) including Nimbin, Azadirachtin, Nimbidin, salannol, salanin, gedunin, quercetin	Pyrolysis and hydrothermal/hydrothermal (i) 180 °C for 2 h/pH = 7.4 200 °C for 12 h	—	HRTEM, AFM, XRD, XPS, FTIR, SAED, EDX, UV-visible spectroscopy, photoluminescence (PL) spectroscopy	2.5/−20.4 mV	–OH, –NH/NH_2_, –COOH, C=O, –CONH_2_, –C–NH_2_
06	Black plum leaves^4,65^ (*Syzygium cumini*)/Myrtaceae	Cancer, diabetes, diarrhea, leucorrhea, stomach pains, increased hemoglobin, skin health improvement	Glycosides, myricetin, ellagic acid, flavonol, caffeic acid beta-sitosterol, alkaloids, flavonoids, steroids, phenols, and tannins	Hydrothermal/180 °C for 2 h/pH = 7.4	O & N groups derived from phytochemicals	HRTEM, FTIR, XPS, UV-Vis spectroscopy, fluorescence lifetime spectroscopy (FLS), zeta potential	3.4/−14.3 mV	–OH, –COOH, C=O, C–O, –NH2, –N–H
07	Jamun fruit^66^ (*Syzygium cumini*)/Myrtaceae	Cough, diabetes, inflammation dysentery, gastrointestinal complaints, ringworm	Quercetin, rutin, ferulic	Hydrothermal/180 °C for 6 h/pH = 5.0	—	HRTEM, DLS, UV-Vis spectroscopy, fluorescence spectroscopy, FTIR, confocal laser microscopy	2.1 ± 0.5/—	–OH, C=O, –COOH, –NH_2_, –CO, C–N, –CHO
08	Pinewood (*Pinus*)^32,67^/Pinaceae/O & N groups derived from phytochemicals	Kidney/liver disorders, cancer, asthma, diabetes, cardiovascular problems, antihypertensive, fungal/bacterial diseases	Alkaloid, tannins, flavonoids, alkaloids, terpenoids cellulose, hemicellulose and lignin	Hydrothermal carbonisation/180 °C for 3 h	O & N groups derived from phytochemicals	HRTEM, AFM, UV-Vis & PL spectroscopy, XPS, FTIR	3.56/—	–OH, –COOH, C=O, C–O–C, –NH
09	Pine needles^47^/Pinaceae	Kidney/liver disorders, cancer, asthma, diabetes, cardiovascular problems, antihypertensive, fungal/bacterial diseases	Alkaloid, tannins, flavonoids, alkaloids, terpenoids cellulose, hemicellulose and lignin	Hydrothermal/200 °C at 6 h/pH = 7.0	Natural C & N source	HRTEM, SEM, DLS, XRD, FTIR, Raman spectroscopy, TGA, UV-Vis spectroscopy	3.56 ± 0.85/—	–OH, C=O, –NH, –COOH, C–O, C–C, C–N, C=C
10	Pinecones and pine bark^5^/Pinaceae	Kidney/liver disorders, cancer, asthma, diabetes, cardiovascular problems, antihypertensive, fungal/bacterial diseases	Alkaloid, tannins, flavonoids, alkaloids, terpenoids, cellulose, hemicellulose and lignin	Microwave pyrolysis at 1000 W for 1 h/pH = 4	—	TEM, HRTEM, SEM, DLS, XRD, FTIR, Raman spectroscopy, TGA, UV-Vis spectroscopy	19.2 (PCCD) 18.39 (PBCD)/—	–OH, –COOH, C=O, C–O–C–CH_2_, –OCH_3_, H–N
11	Tamarind leaves^33,51^ (*Tamarindus indica)* (blue light emitting CDs, b-CQD) & mango leaves (*Mangifera indica*) (green light emitting CQDs; g-CQDs)	Anti-asthmatic, antidiabetic, deep wounds, high fever, malaria, blood related diseases, digestive issues, cardiovascular disorders, anti-venomous, hepato-protective, analgesic, anti-inflammatory and parasitic potentials	Xylose, pectin, glucose, galactose and uronic acid, tartaric acid, tannin, sugars, fiber, protein, carbohydrates Ca, K, Zn, Fe, Na	Microwave: h-CQDS blue-emitting CQDs (b-CQDs) 210 °C for 5 h green-emitting CQDs (g-CQDs) 180 °C for 4 h	—	UV-Vis spectrophotometry, PL spectroscopy, smartphone-based fluorometry, statistical and empirical modeling	—	For g-CQDs: C=C, C=O, C–O–C, R-COOH, RCONH_2_ RCOOR and for b-CQDs: C=C, R-COOH, RCONH_2_, RCOOR
12	Tamarind shell waste^68^ (*Tamarindus indica*)/Leguminoaes	Anti-asthmatic, antidiabetic, deep wounds, high fever, malaria, blood related diseases, digestive issues, cardiovascular disorders, anti-venomous, hepato-protective, analgesic, anti-inflammatory and parasitic potentials	Xylose, pectin, glucose, galactose and uronic acid, tartaric acid, tannin, sugars, fiber, protein, carbohydrates Ca, K, Zn, Fe, Na	Carbonization at 400 °C followed by microwave irradiation for 30 min/pH = 7.0 (CQDs) pH = 12.0 (ZnO)	Nanocomposite CQDs/ZnO	XRD, TEM, HRTEM, FE-SEM, EDX, FTIR, Raman spectroscopy, UV-visible spectroscopy, XPS	40–55 (Photocatalyst)/—	–OH, C–O–C, C–O, C=O
13	Lotus root^35,69^ (*Nelumbo nucifera*)/Nelumbonaceae	Ornamental value; antimicrobial, antibacterial, antifungal, anti-plasmodial, antioxidant, skin infections, reproductive and respiratory diseases	Alkaloids, glutathione, mucilaginous juice, iso-quercetin, amino acids, glucoprotein, polysaccharides, glucoluteolin, starch, asparagines, methanolic extract	Microwave/pH 7.0	N groups derived from phytochemicals	TEM, FTIR, XPS, UV-Vis absorption & fluorescence spectroscopy, fluorescence lifetime measurement	9.41/—	–OH, NH_2_, –C–N, –COOH, C=O, C–H
14	Teak leaves^70,71^ (*T. grandis* Linn.)/Verbenaceae	Bronchitis, hyperacidity, biliousness, leprosy, diabetes, helminthiasis, swellings, astringent paste for wounds	Phenols and phenolic acid, norlignans, flavonoids, anthraquinones, glycosides, alkaloids, terpenoids, apocarotenoids, steroids, lignans	Ultrasonication (at 40 kHz)	—	UV-Vis absorption, FTIR, Raman spectroscopy, XRD, HRTEM, FESEM, EDAX, zeta potential analysis	5/−51.0 mV	–OH, –CH, =C–H, C–O–C, C=O, COO–
15	Tulsi leaves^72,73^ (*Ocimum sanctum*)/Lamiaceae	Common cold/cough/flu (influenza), headache, earache, colic pain, asthma, hepatic diseases, insomnia, malaria fever, ulcer, arthritis, night blindness, digestive disorder, memory enhancer	Eugenol, flavonoids, phenolics, gallic acid, vallinin, terpenoids, oleic acid, palmitic acid, linoleic acid	Hydrothermal, 180 °C for 4 h/pH 7.0	—	FTIR, XRD, TEM, FESEM, UV-Vis absorption, PL spectroscopy, EDAX	3/—	–OH, C=C, C–H, –NH, C–N, –COOH, C=O
16	Tulsi leaves *Ocimum tenuiflorum*^6^			Hydrothermal 200 °C for 12 h	—	DLS, TEM, Raman spectroscopy, FTIR, XPS, SAED, zeta potential, UV-Vis and fluorescence spectroscopy	1–3/−18 mV	–OH, N–H, C=O, COO–, C=C, C–N, C–H, C–O–C
17	Tulsi leaves^74^			Hydrothermal 200 °C for 4 h	—	TEM, EDX, XRD, FE-SEM, TGA, FTIR, zeta potential, UV-Vis spectroscopy, time resolved fluorescence decay	5/−25.7 mV	C=O, C=C, –OH, =N–H, C–H
18	Lily jasmine^75,76^ or motia leaves (*Jasminum sambac*)/Oleaceae	Antiulcer, antioxidant activities, breast cancer, dermatitis, diarrhea, conjunctivitis, fever, asthma, abdominal pain, toothache abscess, uterine bleeding	Flavonoids, alkaloids, phenols, terpenoids, carbohydrates, tannins, proteins, phytosterols, saponins, resin, steroids, salicylic acid	Ultrasonic radiation assisted synthesis	Aq. NH_3_ as N dopant	UV-visible spectrophotometry, FTIR, XRD, EDX, SEM	(i) 22.04/—	–OH, C≡C, C–O, Mn–O
	(i) (CDs-MnO_2_)						(ii) Amorphous	–CH, C=C
	(ii) (NCDs-MnO_2_)							
19	Indian jujube fruit (*Ziziphus mauritiana*)^77,78^/Rhamnaceae	Sedative, hepatoprotective, antimicrobial, hypoglycemic, anti-plasmodial, antidiabetic, anti infectious, analgesic, diuretic, anti-inflammatory and anticonvulsant, useful in old wounds, ulcers diarrhea, antipyretic, anti-obesity, blood purifier, digestion, respiratory disorders	Phenolic compounds flavonoids, alkaloids, terpenoids, lipids, triterpenoic acids, saponin, pectin	Hydrothermal 200 °C for 8 h/pH = 12	Aq. NH_3_ as N dopant	UV-Vis, FTIR, Raman, XRD, XPS, HR-TEM	7.4 ± 1.6/—	–NH, –OH, –C=C, –C–O–C
20	Indian jujube fruit^79^ (*Ziziphus mauritiana* pulp extract)/Rhamnaceae	Sedative, hepatoprotective, antimicrobial, hypoglycemic, anti-plasmodial, antidiabetic, anti infectious, analgesic, diuretic, anti-inflammatory and anticonvulsant, useful in old wounds, ulcers diarrhea, antipyretic, anti-obesity, blood purifier, digestion, respiratory disorders respiratory disorders	Phenolic compounds flavonoids, alkaloids, terpenoids, lipids, triterpenoic acids, saponin, pectin	Hydrothermal 190 °C for 5 h/pH = 7.0	N groups derived from phytochemicals	XRD, UV-visible spectroscopies, SEM, EDX, PL, TEM, FTIR	2.54/−18.7 mV	C–O, C–N, –OH, C–H, N–H, C=C, C=O
21	Mango peels (*Mangifera indica*)^80,81^/Anacardiaceae	Anti-asthmatic, anti-diabetic, deep wounds, malaria, blood-related diseases, digestive issues, cardiovascular disorders, anti-venomous, hepato-protective, analgesic, anti-inflammatory, anti-oxidant, anticancer, anti-microbial	Carotenoids polyphenols, omega-3 and -6 polyunsaturated fatty acids, phytochemicals, micronutrients, fibre vitamins, carbohydrates, proteins, phenolic compound	Hydrothermal, 200 °C for 4 h/pH = 8	Composite of CDs with molecularly imprinted polymers; CQDs@MIPs	FL spectroscopy, FTIR, UV-Vis, TEM, SEM, AFM, XPS, XRD, FL life time	3–5 nm/—	C–H, –OH, =C–O, –COOH, –OH, –NH_2,_ C=O
22	Bael patra fruit^82,83^ (*Aegle Marmelos*)/Rutaceae (i) C-CDs (peel-hard shell) (ii) P-CDs (edible pulp) (iii) M-CDs/(mixture of pulp and gum)	Antioxidant, anticancer, anti-plasmodial, antimicrobial, hepatoprotective activities	Marmelosin, coumarins, marmenol, imperatorin, xanthotoxin, methyl ether, scoparone, umbelliferone, scopoletin, psoralen marmeline	Hydrothermal 180 °C for 12 h/pH = 7.0	Aq. NH_3_	HRTEM, BET, FESEM, FTIR, XPS, UV-Vis spectrophotometry, zeta potential	(i) 3 nm/+0.23 mV	C–N, C=O, =N–H, –OH, –COOH, C–H, C–O
							(ii) 6 nm/+ 0.11 mV	
							(iii) 8 nm/+0.07 mV	
23	Dwarf banana peel^84,85^ (*Musa acuminata*)/Musaceae	Antioxidant, anti-diabetic, immunomodulatory, hypolipidemic, anticancer, anti-microbial properties anti-hypertensive, anthelmintic, tuberculosis, respiratory diseases	Terpenoids, saponins, steroids, alkaloids, anthocyanins, tannins, fatty acids, phenols, steryl esters, sterols, polyphenols, fiber, flavonoids, lignin, lipids, hemicellulose, minerals proteins, cellulose	Hydrothermal/200 °C for 24 h	Aq. NH_3_	XRD, Raman spectroscopy, HRTEM, XPS, ATR-FTIR, PL spectroscopy	4/—	–COO, –OH, C–OH, –COOH C–O–C, –NH_2_, –C–NH
24	Pseudostem of banana^86^ (*Musa acuminata*)/Musaceae	Antioxidant, anti-diabetic, immunomodulatory, hypolipidemic, anticancer, anti-microbial properties anti-hypertensive, anthelmintic, tuberculosis, respiratory diseases	Terpenoids, saponins, steroids, alkaloids, anthocyanins, tannins, fatty acids, phenols, steryl esters, sterols, polyphenols, fiber, flavonoids, lignin, lipids, hemicellulose, minerals proteins, cellulose	Hydrothermal/180 °C for 2 h/pH = 7.4	—	UV-Vis, fluorescence, DLS, FT-IR, Raman, XPS, HRTEM	2.51/—	–OH, C=C, C=O, C–O–C
25	(i) Ripe banana peels^87^ (BCD_BP_)	Antioxidant, anti-diabetic, immunomodulatory, hypolipidemic, anticancer, and antimicrobial properties, anti-hypertensive, anthelmintic, tuberculosis, respiratory diseases	Terpenoids, saponins, steroids, alkaloids, anthocyanins, tannins, fatty acids, phenols, steryl esters, sterols, polyphenols, fiber, flavonoids, lignin, lipids, hemicellulose, minerals proteins, cellulose	Hydrothermal/180 °C for 6 h	(i) Ethylenediamine + l-cysteine	XRD, Raman spectroscopy, HRTEM, XPS, ATR-FTIR, PL spectroscopy	(i) 9.5/—	O–CH_3_, –C–O, OCH_2_, –OH, –C=O, C=C, COOH, C–H, –SH, =NH
	(ii) Dried rose petals (BCD_RP_)				(ii) Ethylene diamine + l-cysteine		(ii) 7.2/—	
	(iii) Biochar							
26	Betel leaves^49,88^ (*Piper betle*)/Piperaceae	Antimicrobial, antioxidants, antifungal, anticancer, anti-inflammatory, anti-diabetic and digestive and gastroprotective characteristics	Calcium, minerals, vitamin C, niacin, carotene, thiamine and riboflavin	Hydrothermal 200 °C for 12 h	N groups derived from phytochemicals	XRD, Raman spectroscopy, TGA, DTA, XPS, TEM, HRTEM, FTIR, UV-Vis and fluorescence spectroscopy	∼4/—	–OH, =NH, C–H, C=O, C=C, C–N, C–OH, C–O–C, –CH_2_, –COOH, C=N, –NH_2_, C–C, C–N–C, –CONH –OH,
27	Betel leaves^88^ (*Piper betle*)/Piperaceae		Alkaloids, flavonoids, tannins, saponins, chavicol, diastase, essential oil	Hydrothermal 180 °C for 12 h/pH = 7	+Ammonia solution (N-dopant)	AFM, HR-TEM, PSA, XPS XRD, FTIR, UV-Vis spectroscopy, fluorescence spectroscopy with lifetime analysis	3.7/—	–NH, –CONH, N–C_3_, C=O, C=N, C–O, C–N, C–N–C
28	Bamboo leaves^89,90^ (*Bambusa vulgaris*)/Poaceae	Paper making, construction (bamboo bridges/railings, living hedges, household items/furniture, tiles *etc.*), medicinal virtues, religious purposes, educational purposes, ornamental purposes and landscape gardening, containers, seed drills	Proteins, amino acids, carbohydrates, minerals, vitamins, phytosterols, fibre, phenols, glycosides	Solvo-thermal 180 °C for 5 h	—	TEM, FTIR, XPS, UV-Vis and fluorescence spectroscopy	3–7/—	C–O, C–N, –OH, C–C, C–H, N–H, C=O
29	Bamboo leaves^91^ branched polyethylenimine (BPEI)-capped CQDs (BPEI-CQDs)	Paper making, construction (bamboo bridges/railings, living hedges, household items/furniture, tiles *etc.*), medicinal virtues, religious purposes, educational purposes, ornamental purposes and landscape gardening, containers, seed drills	Proteins, amino acids, carbohydrates, minerals, vitamins, phytosterols, fibre, phenols, glycosides	Hydrothermal/200 °C for 6 h	—	FTIR, UV-Vis and FL spectroscopy, TEM, zeta potential	3.6/+13.8 mV	COOH, –OH, C=O
30	Pomegranate juice^92,93^ (*Punica granatum* L.)/Lythraceae	Anticancer, stomach disorder, diarrhea, cardiovascular problems, osteoarthritis, diabetes, dental care, anemia	Vitamins (C, K and folate), seed oil, antioxides, polyphenols, ellagitannins, anthocyanins, delphinidin, cyanidin, and pelargonidin glycosides, flavonoids	Hydrothermal 120 °C for 5 h/pH = 5–9	Ammonium hydroxide	UV-Vis absorption, FTIR, HRTEM, XPS	2–5/+ 20.97 mV	=NH, C–H, –OH, COO–, C–OH, C–O–C, C–N
31	Jackfruit seeds^94,95^ (*Artocarpus heterophyllus*)/Moraceae	Antioxidant, anti-inflammatory, antihypertensive, antibacterial, antifungal, anticarcinogenic, antineoplastic; also used in preparation of bakery items breakfast cereals, and infant food	Fibre, proteins, carbohydrates, vitamins, minerals (Mg, K, P, Ca, Na, Fe, C, Zn, Mn) phenolics, flavonoids, terpenoids, steroids, glycosides, saponins, carotenoids, sterol, alkaloids, tannin	Microwave-assisted; 600 W for 1 m & 30 s	—	XRD, TEM, SAED, zeta potential, HRTEM, FTIR, XPS, Raman spectroscopy, UV-vis absorption, PL spectroscopy	5/−43.2 mV	–OH, N–H, C=O, COO–, C=C, C–O
32	Gum Ghatti^96,97^ (a natural exudate from *Anogeissus Latifolia* tree/Combretaceae	Emulsions, suspensions, sizing agent in the paper industry, house construction, treatment of piles, diarrhea, respiratory diseases, liver issues	Tannin, sugars, its nature as a calcium–magnesium salt	Microwave pyrolysis (20 minutes)	—	XRD, XPS, TEM, FESEM, FTIR, UV-Vis spectroscopy, PL spectroscopy	2.6/—	–OH, –CH, C–O, C=C, C=O, C–O–C, C–N
33	Turmeric powder^98,99^ Zingiberaceae	Anti-inflammatory, antiseptic, disinfectant, analgesic, aiding digestion, skin treatment	Alkaloid, curcuminoidflavonoids, amino acids, phenolics, protein	Hydrothermal 200 °C for 24 h	—	XRD, SEM, TEM, FTIR, vibrating sample magnetometer (VSM), UV-Vis absorption, PL spectroscopy	20–100/—	C=O, –OH, C–O, C–H, –COOH
34	(i) Azadirachta indica (NNP)^100^	(i) Therapeutic and biological properties, antioxidant properties	(i) NNP-nimbolide, azadirachtin, ascorbate	Microwave 800 W for 3 min/80 °C for 12 h		XRD, FESEM, EDX, FTIR, UV-Vis spectrophotometry	(i)33/—	(i) C=N, –OH, C–H, C≡N, C–O, C–N
	(ii) Psidium guajava (GuNP)	(ii) Broad health benefits: antioxidant and anti-cancer properties	(ii) GuNP-gallic acid, taxifolin, hesperetin, quercetin, rutin				(ii)32/—	(ii) –OH, C–N, O–H, C≡N, C–H, C=O
	(iii) Holy basil (TNP)	(iii) Facilitates surface functionalization of CNPs and excellent radical scavenging	(iii) TNP-luteolin, apigenin, caffeic acid, rosmarinic acid, quercetin				(iii)42/—	(iii) O–H, C–O, C≡N C≡C, N=C=O
	(iv) Syzygium cumini (JNP)	(iv) Strong oxidant properties	(iv) JNP-quercetin, myricetin, maslinic acid, triacontanol				(iv) 23.5/—	(iv) C=O, O–H, C–N C–O C–H
								(v) C=O N–H, C=N

## 5. Origin of photoluminescence

**Fig. 5:**
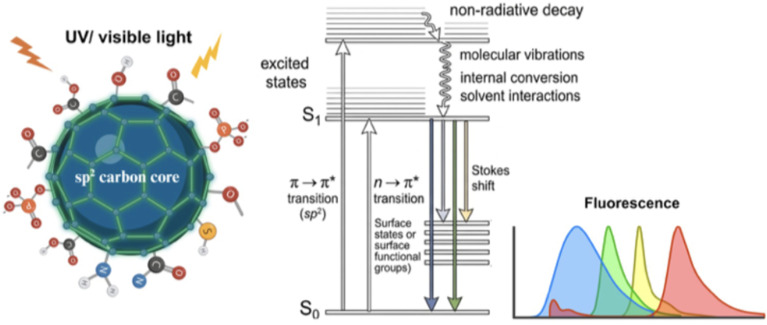
Schematic representation of proposed photoluminescence mechanism in carbon dots.

Although origin of the photoluminescence (PL) mechanism has been vividly explored, it is still not entirely understood owing to the wide variety of precursors and techniques being adopted during the synthesis, exhibiting the formation of a complex system.^101–103^ Hence, a unified theory elucidating for the excitation dependent PL signal has been difficult to establish.^104^ The largely accepted fluorescence mechanisms (schematic representation in Fig. 5) are briefly discussed below.

### 5.1 Quantum confinement effect (QCE)/size effect

Many theoretical researches have correlated the excitation-dependent emission with the quantum confinement in CDs, just as for metal-based quantum dots.^105^ Close to Fermi level, a splitting of energy level occurs, wherein the electronic quasi continuous energy levels get transformed into distinct energy levels leading to widening of energy gap.^106^ According to this phenomenon, when the particle size is brought down to nanometer dimensions, the PL emission is governed by the particle size. In effect, the “core size”/sp^2^ domain size (the effective conjugated length) is the key factor, rather than the actual particle size.^107,108^ An increase in “size” leads to a reduction in band gap, thus shifting the emission wavelength towards red. According to abstract modeling, some of the transitions (π–π*) of sp^2^ clusters lead to the radiative recombination of the excitons within the core, thus affecting the PL of CDs.^109,110^

### 5.2 Surface states

Sun *et al.* were the first to suggest that photoluminescence could be influenced by the surface states in CDs.^103^ Since then, many researchers have supported the relation between the surface states and the functional groups present on the CDs' surface (–COOH, –NH_2_, C=O, C=N, –OH, *etc.*). These groups bring in energy levels (fluorophores) within the energy gap which act as surface energy traps between π and π* of the C–C band. Consequently, this surface modification/passivation with different functional groups eliminates various non-radiative electron–hole recombination centres, stabilizes CDs, and thus enhances their luminescence.^111^

Consequently, the electron transitions from these levels result in a red-shifted emission. Thus, by regulating the surface functional groups (heteroatomic doping), the PL emission behavior can be tailored.^112^ The red-shifted PL emission in CDs is also influenced by the oxygen content present on their surface. The greater the extent of surface oxidation, the higher the proportion of surface defect sites on CDs.^113^ These defects have the ability to capture excitons, the resulting radiation from the recombination of these trapped excitons causes a shift in PL emission towards red leading to a narrower energy band gap (surface-state-related emission).

The excitation dependent emission in most of the biomass-synthesised CDs has been ascribed to be due to different particle sizes (quantum effect) as well as the distributed emissive states, associated with various functional groups, present on CDs' surface. This may be attributed to the discrepancy in the compound structures present in the natural precursors, leading to a molecular transition mode change under different excitations, thus resulting in deviation in emission wavelength.^114^ For example, the green luminescence of the CDs prepared from neem leaves has been attributed to surface energy traps.^64^ However, amine functionalized CDs, (obtained by hydrothermally treating the CDs in ammonia), caused less accumulation resulting in a blue shift in PL emission in contrast to the originally observed green fluorescence. Some of the functional groups present on surface, like –COOH and epoxy, get substituted by –CONH_2_ and –C–NH_2_ after amine functionalization, which represses the non-radiative recombination in the intrinsic states, leading to a twofold magnification in the PL intensity.

### 5.3 Molecular state

In the process of synthesizing CDs *via* bottom-up approach, numerous molecular species (molecular fluorophores with conjugated carbons) are generated. These impurities, either free or attached to CDs' surface, regulate the fluorescence emission, thus influence its overall optical properties^115^

## 6. Detection of heavy metal ions and organic pollutants from IKS-based biomass precursors

The main work describing sensing of organic pollutants and heavy metal ions through IKS based green precursors are highlighted in Table 2. The table incorporates various IKS based natural precursors used in the synthesis of carbon-based sensors as an electrochemical/fluorescent/ratiometric/colorimetric nanoprobes to detect toxic contaminants. Some of the main characteristics used for the assessment of the CDs are quantum yield (QY), linear concentration range (LCR), and limit of detection (LOD), which demonstrate the overall efficiency of the biomass synthesised CDs towards the sensing of the analyte. Additionally, recovery and relative standard deviation observed in the presence of real samples have also been highlighted.

**Table 2:** Plant biomass used as precursors for optical sensing of heavy metal ions and organic pollutants^a^

S. no.	Precursor/dopant	Maximum PL emission wavelength, *λ*_max_, (excitation wavelength, *λ*_ex_)/QYs	Analyte	Detection type/degradation approach	Selectivity/sensitivity	Repeatability/recovery	Relative standard deviation (RSD)
01	Curry leaves extract and gold NPs (AuNPs) electrode on graphite sheet substrate^3,26^	—	Heavy metal ions: (i) Pb(ii) (ii) Hg(ii)	Electrochemical sensing	Pb(ii), Hg(ii)	—	—
02	Giloy stem/N, S doping^28,46^	520 nm (430 nm)/7.2%	Nitrophenol and dye: (i) 4-NP; (ii) CR	(i) Fluorometric quenching: IFE; LOD: 380 nM	(i) 3-NP, 4-NP, 2-NP, phenol, 1-naphthol, P-BQ, P-PDA, CNB, and biomolecules	(i) 4-NP (a) for tap water: —/94.67 to 103.45% (b) for pond water: —/93.88 to 102.49%	(i) 4-NP (a) for tap water: ≤3.96% (*n* = 3) (b) for pond water ≤4.38% (*n* = 3)
				(ii) Fluorometric quenching: IFE; colorimetric and smartphone techniques; LOD: 62 nM	(ii) CR, Ba(ii), Ca(ii), Cd(ii), Cu(ii), Mg(ii), Mn(ii), Na(i), K(i), Zn(ii), Cl^−^, NO_3_^−^, dyes, molecules (MB, aniline, MG, naphthol)	(ii) CR (a) for tap water: —/95.61 to 99.34% (b) for pond water: —/95.05 to 96.4%	(ii) CR (a) for tap water, ≤4.92 (b) for pond water 4.54% (*n* = 3)
03	“Shatavari” *Asparagus racemosus* root^27,48^	447 nm (216 nm)/—	Heavy metal ion: Ag(i)	Colorimetric: (i) CD color change from yellow to brown due to interactions between CDs and Ag(i); (ii) change in the maximum absorption wavelength (370 nm to 454 nm)	Ag(i), K(i), Na(i), Ca(ii), Cd(ii), Pb(ii), Zn(ii), Hg(ii), Mg(ii), As(v)	—	—
04	(i) Neem seed/aqueous NH_3_ (as N source)^50,63^	400 nm (320 nm)/—	Dye: Safranin-O	Reduction of dye with NaBH_4_ using CDs (6 min)	Safranin-O dye	—	—
05	Neem leaves + ammonia solution amine-terminated GQDs (Am-GQDs)^30,64^	—/2%	Heavy metal ion: Ag(i)	Fluorometric quenching original fluorescence regenerated using l-cysteine. LOD: 0.033–0.1 g L^−1^	Ag(i), Ni(ii), Cu(ii), Co(ii), Fe(ii), Hg(ii), Fe(iii), Pb(ii) can quench the fluorescence (off-state), only Ag(i) allows for the fluorescence recovery (on-state) upon l-cysteine addition	—	—
06	Jamun leaves^4,65^ (O & N groups derived from phytochemicals)	460 nm (330 nm)/15.9%	Heavy metal ion: Fe(iii)	Fluorometric quenching LOD: 0.13 µM LCR: 0–80 µM	Fe(iii), Na(i), Mg(ii), K(i), Pb(ii), Ca(ii), Mn(ii), Zn(ii), Cu(ii), Cd(ii), Ni(ii), Co(ii), Ba(ii), Al(iii), Cr(iii)	—/88 to 106.5%	0.649 to 1.272%
07	Jamun fruit^66^	438 nm (350 nm)/5.9%	Heavy metal ion: Fe(iii)	Fluorometric quenching and absorbance LOD: 0.001 µM LCR: 0.01–100 µM	Fe(iii), Mg(ii), K(i),Ca(ii), Hg(ii), Mn(ii), Fe(ii), Co(ii), Pb(ii), Zn(ii), Ba(ii), Cr(iii), Al(iii)	—/94.0 to 107.3%	0.03 to 1.63%
08	Pinewood^32,67^	447 nm (330 nm)/4.69%	Heavy metal ion: Fe(iii)	Fluorometric LOD: 355.4 nM L^−1^ LCR: 0–1000 µM L^−1^ and 1000–2000 µM L^−1^	Fe(iii), K(i), Cd(ii), Fe(ii), Ag(i), Mg(ii), Zn(ii), Mn(ii), Sn(ii), Hg(ii), Pb(ii), Cu(ii)	—	—
09	Pine needles*/*natural C & N source^47^	400 nm (320 nm)/7.65%	Heavy metal ion and food additives: (i) Fe(iii) (ii) folic acid (FA)	Dual-mode: fluorometric and UV-Vis absorbance (i) Fe(iii) LOD-0.04 µM (UV-Vis mode); 0.02 µM for the fluorescence mode) LCR: 0.1–540 µM; (ii) folic acid LOD-0.04 µM (fluorescence mode) 0.03 µM (UV-Vis mode); LCR: 0.1–165 µM	(i) Fe(iii), Cd(ii), Na(i), Mg(ii), Ba(ii), Ni(ii), Cu(ii), Li(i), Hg(ii), Pb(ii), K(i), Co(ii), Ca(ii), Zn(ii), Fe(ii)	(i) For Fe –/98.66 to 102.46%	(i) ≤ 1.78%.
					(ii) l-glutathione (GSH), l-cysteine (cys), glucose (glu), dopamine (DA), dl-homocysteine (Hcy), nicotinic acid (NA), ascorbic acid (AA), taurine (TA)	(ii) For FA –/96.00 to 102.00%	(ii) 1.95%
10	Pinecones (PC) and pine bark^5^ (PB)	PCCD: 430 nm (360 nm)/11.3%	Antibiotics: (i) tetracycline (TC) (ii) amoxicillin (AMX)	Fluorometric quenching: IFE (TC); SQ(AMX); (a) LOD for TC 0.062 µM for PCCDs (b) 0.2237 µM for PBCDs (ii) AMX LOD: 0.49 µM for PBCDs LCR: 5–100 µM	Amoxicillin (AMX), tetracycline (TC), Zn(ii), Fe(iii), Cd(ii), Fe(ii), Hg(ii), Ni(ii), chloramphenicol (CLM), ciprofloxacin (CIP), sulfamethazole (SMZ)	(i)TC —/96.12 to 102.74%	(i) TC <2%
		PBCD: 430 nm (345 nm)/5.64%				(ii) AMX —/98.72 to 100.84%	(ii) AMX <1%
11	Tamarind leaves^33,51^ blue emitting CQDs (b-CQD) & mango leaves green emitting CQDs (g-CQD) both CQDS combined to form hybrid CQDs (h-CQDs)	b-CQDs – blue emission/365 nm	Heavy metal ion: Hg(ii)	Ratiometric fluorescence	—	—	—
		g-CQDs – green emission/365 nm					
12	Tamarind shell waste^68^ CQDs/ZnO nanocomposite	—	Dyes: MB, MG	Dye degradation with CDs-ZnO under solar light exposure. 100%/60 min	—	—	—
13	Lotus root^35,69^/nitrogen content of 5.23%	435 nm (360 nm)/19%	Heavy metal ion: Hg(ii)	Fluorometric quenching; SQ/PET; LOD: 18.7 nM; LCR: 0.1 to 60.0 µM	Hg(ii), Mg(ii) Cd(ii), Cu(ii), Pb(ii), Sr(ii), Fe(iii), Ca(ii), Al(iii), Ba(ii), Co(ii), Fe(ii), Zn(ii)	—/90.0 to 98.5%	—
14	Teak leaves^71^	—	Dye: MB	Adsorption of MB onto CDs (adduct formed between MB^+^ dye and TBCDs^−^) removal rate – 50.1%	—	Yes	—
15	Tulsi leaves^72^	500 nm (450 nm)/9.3%	Heavy metal ion: Pb(ii)	Fluorometric quenching: Pb(ii) LOD: 0.59 nM; LCR: 0.01–1.0 µ M	Pb(ii), Ni(ii), Co(ii), Cu(ii), Cd(ii), Hg(ii), Ca(ii), Mg(ii), Sn(ii), Na(i), K(i), Al(iii)	—	—
16	Tulsi leaves^6^	405 nm (320 nm)/11.5%	Food additive: MG	Fluorometric quenching: both SQ and DQ: LOD 18 nM; LCR: 0.018–0.063 µM	—	—	—
17	Tulsi leaves^74^	435 nm (360 nm)/3.06%	Heavy metal ion: Cr(vi)	Fluorometric quenching: IFE + SQ; LOD: 4.5 ppb; LCR: 1.6–50 µM	Cr(vi), I^−^, NO_3_^−^, H_2_PO_4_^−^, HSO_4_^−^, Br^−^, Cl^−^, F^−^, SO_4_^−^, CN^−^, CH_3_COO^−^, IO_4_^−^/yes, can be used multiple times	—/93 to 99%	
18	Lily jasmine (aq. NH_3_ as N dopant)^75,76^		Heavy metal ion: Cr(vi)	UV-Vis spectrophotometric method (i) for (CDs-MnO_2_), LOD: 16 µM LCR:1–30 µM (ii) for (NCDs-MnO_2_) LOD: 69 µM LCR: 7200 µM	Cr(vi), Hg(ii), Co(ii), Cu(ii), Fe(iii), OH^−^, Cl^−^, NO_3_^−^, SO_4_^2−^	(i) —/100.01 to 100.2%	
	(i) (CDs-MnO_2_)					(ii) —/99.9 to 100.01%	
	(ii) (NCDs-MnO_2_)						
19	“Indian jujube” *Ziziphus mauritiana* fruit)^77,78^/aq. ammonia (N-CDs)	547 nm (370 nm)/—	Dye: safra nin-O (under the presence of NaBH_4_)	N-CDs as catalyst for dye removal: removal efficiency −79% (18 min)	—	—	—
20	*Ziziphus mauritiana* pulp extract^79^/nitrogen-doped (N groups derived from phytochemicals)	450 nm (340 nm)/30%	Antibiotics: CIP	Fluorometric enhancement: PL enhancement attributed to complex formed between the amine group (CDs) and the carboxylate group (CIP) LOD: 0.56 µM LCR: 10–100 µM	Ciprofloxacin (CIP), gentamicin, ampicillin, amoxicillin, tetra-cycline, moxifloxacin, levofloxacin, and norfloxacin	—/92.7 to 101.50%	0.48 to 5.68%
21	Mango peels^80^ (composite of CDs with molecularly imprinted polymers; CQDs@MIPs)	453 nm (360 nm)/—	Pesticide: mesotrione	Fluorometric quenching: SQ and electron transfer LOD: 4.7 nM L^−1^ LCR: 0.015 to 3.0 µM L^−1^	Vitamin B5, vitamin B2, Zn(ii), Ca(ii), Fe(iii), Na(i), Mg(ii), K(i) leucine, glutamate, histidine, alanine, valine, glycine, isoleucine, arginine, threonine, aspartate, phenylalanine, proline, tryptophan, serine, methionine, tyrosine, lysine, fructose, glucose sucrose	—/91.4 to 96.2%	3.2 to 6.1%
22	Bael patra fruit^82^	(i) 431 nm(330 nm)/17.39%	Food additive and heavy metal ion: (a) Allura red; (b) Fe(iii)	Fluorometric quenching: DQ LOD for: (a) Allura red (i) 0.607 µM, (ii) 0.260 µM (iii) 0.166 µM LCR-0–30 µM (b) Fe(iii) (i) 0.144 µM (ii) 0.142 µM (iii) 0.164 µM LCR: 0–150 µM	(i) Allura red, Congo red, erythrosine extra bluish, trypan blue, amaranth, Nile red, bromophenol blue, Sudan I, (ii) Fe(iii), Ag(ii), Ba(ii), Bi(ii), Co(ii), Cu(ii), Hg(i) K(i), Li(i), Mg(ii), Ni(i), Pb(ii), Ca(ii), Cd(ii) and different l-amino acids *i.e.* aspartic acid, asparagine, glutamine, tyrosine, leucine, isoleucine, histidine, alanine, arginine, cysteine, glutamic acid, valine and glycine lysine, methionine, phenylalanine, proline, serine, threonine, tryptophan	—/89.41 to 96.83%	—
	(i) C-CDs (peel, hard shell)	(ii) 433 nm (350 nm)/59.07%					
	(ii) P-CDs (edible pulp)	(iii) 449 nm(370 nm)/55.25%					
	(iii) M-CDs (mixture of pulp and gum)						
23	(i) Dwarf banana peel^84^*/*(+aq. NH_3_)	413 nm (345 nm)/23%	Heavy metal ion: Fe(iii)	Fluorometric quenching: Electron/energy transfer (ET) between Fe(iii) and HN-CDs (non-radiative) LOD-0.66 µM; LCR-5–25 µM	Fe(iii), Ca(ii), Al(iii), Cd(ii), Cr(iii), Co(ii), Cu(ii), Mn(ii) Hg(ii), Zn(ii), Pb(ii), Ni(ii)	—	—
24	Pseudo-stem of banana^86^	—/48%	Heavy metal ion Fe(iii) and S_2_O_3_^2−^	Fluorometric quenching: DQ (electron transfer from CDs to Fe(iii)) (i) for Fe(iii): LOD 6.4 nM; LCR 0–100 µM (ii) for S_2_O_3_^2−^ LOD 0.847 µM	Fe(iii), S_2_O_3_^2−^, Ag(i), Co(ii), Mn(ii), Fe(ii), Cr(iii), Cu(ii), Al(iii), Pb(ii), Mg(ii), Ni(ii), Zn(ii), Hg(ii), Ca(ii), Cd(ii), “turn-on” property towards S_2_O_3_^2−^ anion	—	—
25	(i) Ripe banana peels^87^ (BCD_BP_) + ethylene diamine + l-cysteine	(i)437 nm (330 nm)/27%	Heavy metal ions (i) Fe(iii) (ii) Cr(vi) (iii) dyes: MB, MG, MO and RB	Fluorometric quenching: (i) LOD: 121 pM LCR: 10–200 pM (ii) LOD: 81 pM LCR:10–100 pM (iii) adsorption for dye removal (>90% of dye removal)	(i) Fe(iii), Cr(vi), Mg(ii), Cu(ii), Na(i), K(i), Hg(ii), Cd(ii)	(i) –/94.5 to 100.26%	(i) 0.30–1.45
	(ii) Dried rose petals (BCD_RP_) + ethylene diamine + l-cysteine	(ii) 407 nm (316 nm)/28%			(ii) Fe(iii), Cr(vi), Hg(ii), Mg(ii), Cu(ii), Na(i), Cd(ii), K(i)	(ii) –/87.0 to 102.3%	(ii) 0.27–1.23
	(iii) Biochar				(iii) —	(iii)—	(iii)—
26	Betel leaves^49^ N-doped CDs (betel leaves acted as C & N source)	428 nm (360 nm)/12%	Heavy metal ion: Fe(iii)	Fluorometric quenching; resonance energy/electron transfer (RET) mechanism resulting in CDs + Fe(iii) adduct LOD 0.43 µM, LCR 5–30 µm	(i) Fe(iii), Al(iii), Cd(ii), Ca(ii), Co(ii), Cu(ii), Cr(iii), Hg(ii), Ni(ii), Mn(ii), Zn(ii), Pb(ii)	—	—
27	Betel leaves^116^ +ammonia solution (green N-dopant)	402 nm (320 nm)/4.21%	(i) Food additive: Picric acid (PA)	Enhancement in absorbance & Fluorometric quenching; (i) for PA LOD-0.11 µM LCR: 0.3–3.3 µM (ii) for Fe(iii) LOD: 0.135 µM; LCR: 0.3–3.3 µM	(i) Picric acid phenol, nitrobenzene, aniline, hydroquinone, 4-nitrophenol, benzo- quinone, benzoic acid, toluene	—	—
			(ii) Heavy metal ion Fe(iii)		(ii) Fe(iii), K(i), Cu(i), Na(i), Pb(ii), Cu(ii), Cr(vi), Zn(ii), Fe(ii), Cd(ii), Ag(ii), Hg(ii) Mn(ii)		
28	Bamboo leaves + anhydrous ethanol^89^	(i) 493 nm & 653 nm (400 nm)/4.7%	Heavy metal ions: (i) Pb(ii); (ii) Hg(ii)	Ratiometric fluorescence (i) for Pb(ii): LOD: 0.14 nM; LCR: 0.6–800 nM (ii) for Hg(ii): LOD: 0.22 nM; LCR: 1–1000 nM	(i) Pb(ii) Al(iii), Cr(iii), Fe(iii), Cu(ii), Mg(ii), Zn(ii),Hg(ii), Ca(ii), Mn(ii), Cd(ii), Co(ii), Ag(i), K(i),Na(i)	(i) –/91.4 to 105.6%	(i) 1.4 to 5.7% (*n* = 6)
	(i) Dual-emission CD nanohybrids	(ii) 491 nm, 611 nm, 665 nm (400 nm)/3.8%			(ii) Hg(ii), Al(iii), Pb(ii) Fe(iii), Cu(ii), Cr(iii), Mg(ii), Ca(ii), Zn(ii), Cd(ii), Mn(ii), Ag(i), Co(ii), K(i),Na(i)	(ii) 95.3 to 105%	(ii) 4.1–6.9% (*n* = 6)
	(ii) three-emission CD nanohybrids obtained with Na_2_CO_3_ solution						
29	Bamboo leaves^91^ branched polyethylenimine (BPEI)-capped CQDs (BPEI-CQDs)	440 nm (365 nm)/7.1%	Heavy metal ion: Cu(ii)	Fluorometric quenching: IFE; LOD: 115 nM; LCR: 0.333 to 66.6 M	Cu(ii), Co(ii), Mn(ii), Ca(ii), Ni(ii), Hg(ii), Cd(ii) Pb(ii),Ba(ii)	—	—
30	Pomegranate juice^93^ + ammonium hydroxide (as the nitrogen doping agent)	395 nm(310 nm)/—	Nanoparticles: Silver nanoparticles (AgNPs)	Fluorometric quenching: IFE; LOD: 3.8 × 10^−10^ M; LCR: 8.3 × 10^−10^ – 3.3 × 10^−8^ M	AgNPs, Ag(i), Fe(ii), Al(iii), Na(i), Mg(ii), Ni(ii), Cu(ii) Zn(ii)	—/96 to 105%	3.3–4.3%
31	Jackfruit seeds^95^	437 nm (360 nm)/17.91%	Heavy metal ion: Au(iii)	Fluorometric quenching: (ground state complex formation) LOD: 239 nM; LCR: 0–100 µM	Na(i), K(i),Ca(ii), Mn(ii), Fe(ii), Fe(iii), Co(ii), Cu(ii), Zn(ii), Ag(i), Hg(ii), Pb(ii), Au(iii)	—	—
32	Gum Ghatti^97^ C_ZnO_-dots (CZ-2) Zn supported carbon dots	465 nm(370 nm)/—	Dye: MG	Photo-catalytic activity of the C_ZnO_-dots (CZ-2). Degradation efficiency = 98.4%/60 min	—	3 times/—	
33	Turmeric powder^99^/CoFe_2_O_4_; CoFe_2_O_4_-CD nanocomposite	565 nm (330 nm)/20%	Azo dyes: (i) acid black 24 (ii) acid Brown 14 (iii) acid red	Photo-catalytic activity of the CoFe_2_O_4_-CD nanocomposite	—	—	—
34	(i) Azadirachta indica (NNP)^100^	(i) —/21%	Heavy metal ions: (i) Fe(ii) (ii) Ni(ii) (iii) Fe(ii) Pd(ii) (iv) Fe(iii)	Fluorescence quenching (i) DQ, LOD: 0.11 ppm (ii) SQ + DQ, LOD: 0.09 ppm (iii) DQ, 0.011 ppm and 0.0225 ppm (iv) DQ, LOD: 0. 012 ppm)	(i) Fe(ii), K(i), Pb(ii), Cu(ii), Ni(ii), Zn(ii), CO(ii), Ca(ii), Fe(iii), Mg(ii), Sr(ii) Al(iii), (ii) Ni(ii) K(i), Pb(ii), Cu(ii), Zn(ii), CO(ii), Ca(ii), Mg(ii), Fe(ii), Fe(iii), Al(iii), Sr(ii) (iii) Fe(ii), Pb(ii), K(i), Al(iii), Cu(ii), Ni(ii), Zn(ii), CO(ii), Ca(ii), Mg(ii), Fe(iii), Sr(ii) (iv) Fe(iii), K(i), Pb(ii), Cu(ii), Ni(ii), Zn(ii), CO(ii), Ca(ii), Mg(ii), Fe(ii), Fe(iii), Al(iii), Sr(ii)	—	—
	(ii) Psidium guajava (GuNP)	(ii) —/17.8%					
	(iii) Holy basil (TNP)	(iii) —/25.2%					
	(iv) Syzygium cumini (JNP)	(iv) —/19.3%					

a Nitrophenol – NP, Congo red – CR, malachite green – MG, methylene blue – MB, Rhodamine B – RB, methyl orange – MO, ciprofloxacin – CIP.

In a specific study conducted with Jackfruit seeds^95^ as a biomass precursor to synthesize N-doped CDs *via* microwave-assisted method, *O*-phosphoric acid (mild acid) was added to enhance the absorption of microwave radiation that led to passivation of the surface. On the other hand, as an example of multimode detection approaches, in Giloy stem based CDs,^46^ the authors also developed a smartphone-based sensing of the Congo red (CR) dye, apart from the colorimetric and fluorescence techniques, with high-definition images accompanied by the linear relationship of CR with color intensity. In another study, researchers produced hybrid CDs (h-CDs) from the leaves of *Tamarindus indica* and *Mangifera indica*^51^ to develop ratiometric fluorescence sensor. The authors studied the color change of the PL intensity ratio of the h-CDs (obtained from blue (b) and green (g) emitting CDs) with the addition of Hg(ii), the volume ratio of the two CDs and the duration of microwave irradiation. The Hg(ii) detection sensitivity was observed highest for 2 : 1 ratio of b-CDs to g-CDs with microwave irradiation time of 15 minutes.^51^

Furthermore, in a detailed adsorption study with teak leaves biomass carbon dots (TBCDs), the authors evaluated efficiency of the CDs with MB dye and concluded the formation of an adduct between the two, which possessed a significant adsorption capacity of 735.2 mg g^−1^ and removal efficiency of 50.1%. The 77.1% of the adsorbent's efficiency was restored after regeneration, making them effective for reuse.^71^

Using extracts of the bamboo leaves as the natural biomass material, two different types of multi-emission CD hybrids (dual-emission and three-emission CDs) were synthesised by solvothermal means under neutral and alkaline mediums, respectively. Further, these were employed as different ratiometric fluorescent nanosensors for the detection of Pb(ii) and Hg(ii), under ideal and real (river water) samples. The authors observed that the dual-emission CD (for the detection of Pb(ii)), exhibited a linear positive correlation of I_653_/I_493_ with increasing Pb(ii) concentration, while for the three-emission nanosensor, the ratio I_611_/I_491_ decreased linearly with increasing Hg(ii) concentration, in artificial water samples. This indicated categorical Pb(ii) and Hg(ii) level-dependent ratiometric responses for both the CDs. The results confirmed that the ratiometric fluorescent nanosensors not only possessed exceptional sensitivity, selectivity and high precision, but also displayed built-in correction of external effects.^89^

For finding an ideal environment for the detection of Cr(vi) using jasmine leaves^75^ synthesised CDs, the authors studied different factors like concentration of ions, pH, temperature, reaction time, and interfering species. The authors deduced the optimum pH for CDs-MnO_2_ and NCDs-MnO_2_ nanocomposites as 4 and 6 respectively, revealing requirement of more acidic environment for CDs-MnO_2_. Further, the optimum reaction temperature and time interval for CDs-MnO_2_ & NCDs-MnO_2_ nanocomposites required for Cr(vi) detection was 65 °C & 45 °C and 4 min & 7 min, respectively. Further, both the nanocomposites exhibited interference with nitrate ions. Finally, they found NCDs-MnO_2_ nanocomposite to be a better sensor of Cr(vi), attributing to its smaller size and the effective surface functional groups present.

The fluorescence quenching mechanism in tulsi leaves derived CDs for the detection of Cr(vi) was explained to be due to IFE. The authors demonstrated (three times), a significant recovery of the fluorescence by the addition of ascorbic acid which was ascribed to be due to the conversion of Cr(vi) to Cr(iii), allowing the system to be recycled. Further, these CDs were used to sense Cr(vi) in real water samples (industrial water) with satisfactory results.^74^ As another example, nitrogen doped CDs synthesised hydrothermally from betel leaves acted as a single probe system for dual sensing (picric acid, PA and Fe(iii)) with remarkable sensitivity and selectivity. The fluorescence in these CDs displayed great stability even at high ionic strengths. The fluorescence quenching and the red shift (slight) of the emission spectrum with increasing PA concentration was explained to be due to the electrostatic interaction between the O & N based groups (CDs) and the phenolic group of analytes (PA). Likewise, the quenching observed with Fe(iii) was explained to be due to chelation formation, leading to electron transfer from excited state of N-CDs to d-orbital (partially filled). This facilitated non-radiative recombination of electrons & holes, thus diminishing the fluorescence.

The N-CDs synthesised from *Ziziphus mauritiana*, were employed as a catalyst for the removal of Safranin-O (SO) dye, with NaBH_4_ being used as a reducing agent. The authors achieved 79% degradation efficiency of the dye using N-CDs (at pH 12) in 18 minutes as compared to 48% (210 min) with NaBH_4_ only. This was ascribed to the transfer of electrons from BH_4_^−^ ions to the dye, with N-CDs acting as mediators.^78^ In another study, reduction of SO dye was acheived by nitrogen doped CDs derived from neem seeds, which showed a 100% reduction in 6 minutes as compared to 28 minutes in undoped CDs.^50^ Dwarf banana peel^84^ synthesised CDs (doped with aqueous ammonia) as well as betel leaves^49^ derived-NCDs can also be used as an invisible ink, in anticounterfeiting applications.

## 7. Underlying mechanisms for CDs-based optical sensors

**Fig. 6:**
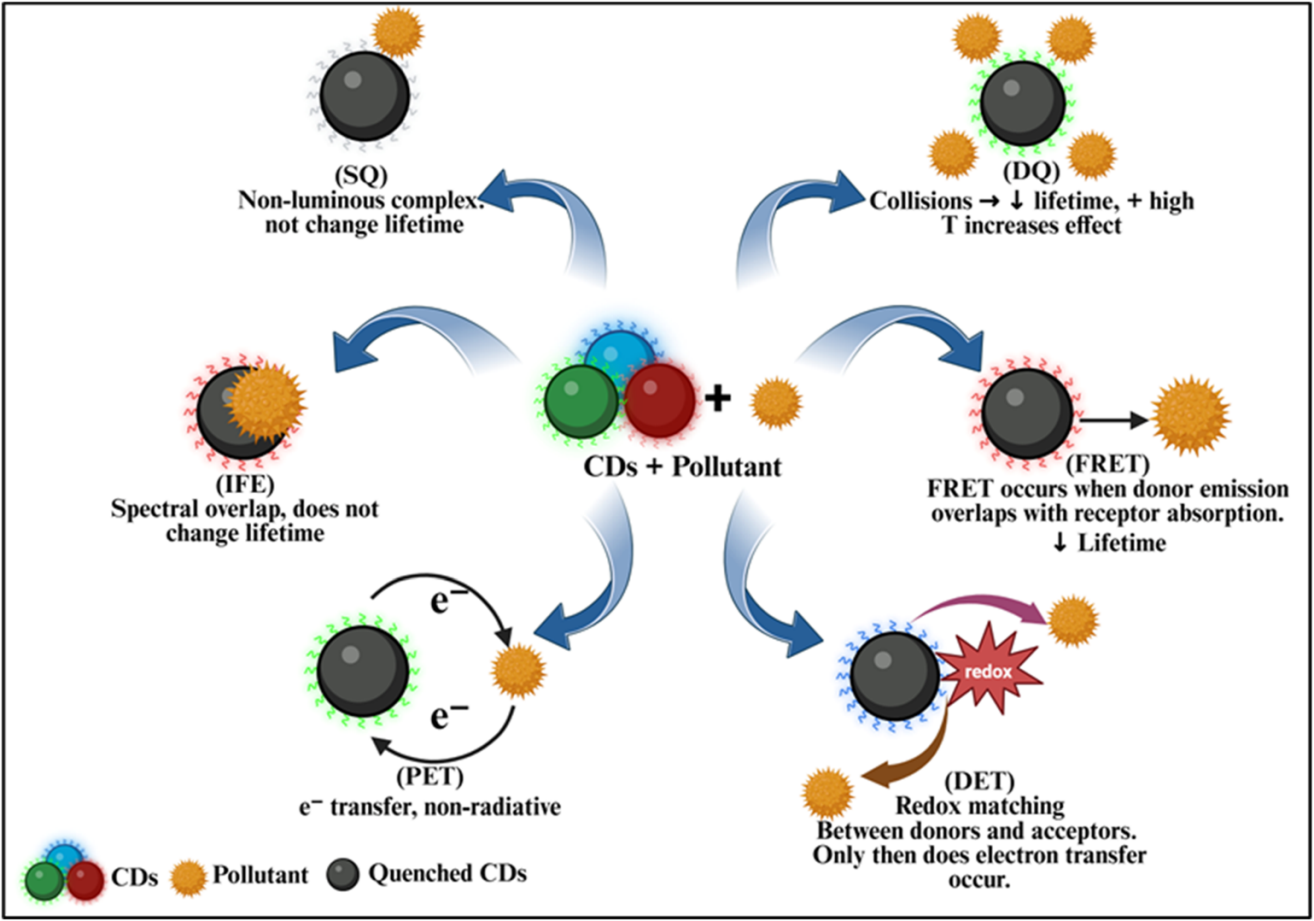
Proposed detection mechanisms in CDs based optical sensors.

In general, an interaction between CDs and analytes (organic/inorganic) results in spectral shift (blue/red/null) accompanied by either quenching or enhancement of their fluorescence intensity. This principle forms the basis for employing CDs as a sensor for detecting these analytes. Depending on the interactions between the CDs and the analytes, the quenching mechanism could be either, (i) static quenching (SQ) or dynamic quenching (DQ); (ii) Inner Filter Effect (IFE) or Foerster resonance energy transfer (FRET); (iii) Dexter energy transfer (DET) or Photoinduced electron transfer (PET) (Fig. 6).^117^

In static quenching (SQ), a weak bonding between the CDs (fluorophore) at ground state and the quencher results in the formation of a non-luminous complex. In contrast, dynamic quenching (DQ) results because of the collision between the CDs and the quencher molecules involving charge/energy transfer, which leads to the de-excitation of CDs and hence reduction in PL intensity.^118^ However, there are few remarkable differences between the two phenomena: in SQ, the luminous lifetime of CDs remains the same (before and after being exposed to the quencher), whereas the absence/presence of quenchers in the detection system affects (shortens) PL lifetime in the case of DQ. In addition, for static quenching, the ground-state complex formation can cause observable changes in absorption spectrum of CDs (shift in peak position/increased number of UV absorption peaks *etc.*) while no change in absorption spectra is detected for DQ. Further, an increase in temperature minimizes the SQ effect, whereas vigorous collisions cause an escalation in DQ effect under high temperature conditions.

The IFE occurs when the UV-visible absorbance spectra of quenchers interfere or overlap spectrally with CDs' fluorescence (FL) excitation or emission spectra, which results in quenching of the PL intensity.^119^ This effect eliminates any interaction between the CDs and the quencher. In most cases, IFE does not cause any variation in CD's absorption spectrum, implying that no new substance is being formed. Hence, the presence or absence of the quencher does not impact CD's average PL lifetime. Whereas in FRET, an overlap of the donors' (CD's) emission spectrum and the acceptor's (quencher's) absorption spectrum occurs, resulting in non-radiative energy transfer between CDs in the excited state and quencher in the ground state (involving dipole–dipole interaction between the two). This transfer is highly effective when the distance between the donor and the receptor is within 10 nm. Also, the PL lifetime of fluorophores (CDs) decreases after their interaction with quenchers in the case of FRET.^120^

The PET sensing mechanism involves formation of complex between CDs and the target analyte. Irradiation with light causes excitation of electrons in CDs. When the transfer of electrons occurs from activated CDs (electron donor) to the quenchers (electron receptor), the process is termed as oxidative PET. On the other hand, the electron transfer from the analyte/quencher (electron donor) to activated CDs (electron acceptor) is named as reductive PET. The deexcitation of electrons, causes non-radiative luminescence and thus changes the PL intensity.^120^ DET is another process wherein a matching of the redox potentials of donors and acceptors is essential for the electron transfer to take place.

The CDs have also been employed in the photo induced degradation of organic dyes. In addition, CDs with metal oxides/semiconductors (composites) having vast surface area as well as exceptional absorption capacity, act as hybrid photocatalysts promoting e-h separation. This further expedites the transfer of electrons, escalating the reduction/oxidation capability of generated carriers, leading to enhanced photo decolorization^121,122^

The PL enhancement-based detection of Ciprofloxacin (CIP) from the green CDs synthesized from *Ziziphus mauritiana* pulp has been reported based on the complex formation between the amine group from CDs and the carboxylate group of CIP.^79^ The PL enhancement was attributed to several factors: (a) transfer of electrons from CDs to CIP; (b) interactions between the functional groups of the analyte and CDs through hydrogen bonding; (c) the reduction of surface defects of CDs' in the presence of CIP.

On the other hand, a colorimetric sensor is an elementary device used to assess an analyte by observing/measuring the color resolution (the depth of color)/change.^120^ The mechanism responsible for the color change in carbon dot-based colorimetric sensor involves special interactions between carbon dots and the target analytes which impact CDs' optical properties. As an example, when AgNO_3_ is exposed to CDs solution, the crucial interactions between the oxygen containing functional groups (hydroxyl, carboxyl and carbonyl), present on the surface of CDs help in enabling electron transfer efficiently to silver ions, leading to reduction of AgNO_3_ to form silver nanoparticles (AgNPs) and thus act as sensors.^48^

Furthermore, designing of the ratiometric fluorescent probes is based on the relative change observed in fluorescence intensity of the emission peaks with the addition of analytes.^123^ The ratio of the PL intensity at two different wavelengths is found to be directly proportional to the analyte concentration, thus helps in setting a coherent self-calibration system capable of eliminating false signals. Hence, this method is more accurate, sensitive as well as more resistant to environmental disturbances and instrumental settings. For example, to detect Hg(ii) in pure and simulated seawater, ratiometric fluorescence assay between blue carbon quantum dots (b-CQDs) (produced from *Tamarindus indica* leaves) and green carbon quantum dots (g-CQDs) (produced from *Mangifera indica* leaves) was employed to synthesize hybrid carbon quantum dots (h-CQDs).^51^ An increase in the PL intensity ratio was observed with increasing mercury ion concentration and hence it could be used to assess the change in color (blue to green) with the addition of Hg(ii).

## 8. Summary and outlook

In conclusion, carbon dots and their composites synthesized using plants from Indian knowledge system represent a sustainable and versatile platform with promising applications in environmental remediation. The vast diversity of natural compounds (plant metabolites/phytochemicals) present in these plants provides a distinctive strength for tuning the properties of carbon dots. However, issues of repeatability/precision due to variability in biogenic substances, mechanistic understanding at molecular level, potential toxicity, and mass production remain among the main challenges to be addressed in the near future. The fundamental understanding of the connection between the phytochemicals present in the green precursor and the characteristics of the CDs after processing is necessary for the design and synthesis of predictable carbon-based nanomaterials. In addition, due to the vastness of IKS, integrating traditional plant-based resources with advanced approaches/techniques for developing standardized carbon dots synthesis protocols is also essential. Simultaneous evaluation of long-term ecological impacts is also crucial for creating large-scale, viable options. Through collaborative and systematic efforts from researchers in the field of biology, chemistry, materials science, medicine and engineering, plant-mediated carbon-based materials derived from India's vast biodiversity hold tremendous opportunity to advance the field of green nanotechnology and make significant to global sustainable development.

## Conflicts of interest

There are no conflicts to declare.

## Data Availability

This review does not generate new data. The data used for analysis and discussion are derived from previously published studies, which are appropriately cited throughout the manuscript.
